# Colistin Resistance in Monophasic Isolates of *Salmonella enterica* ST34 Collected From Meat-Derived Products in Spain, With or Without CMY-2 Co-production

**DOI:** 10.3389/fmicb.2021.735364

**Published:** 2022-01-06

**Authors:** Xenia Vázquez, Vanesa García, Javier Fernández, Margarita Bances, María de Toro, Víctor Ladero, Rosaura Rodicio, M. Rosario Rodicio

**Affiliations:** ^1^Área de Microbiología, Departamento de Biología Funcional, Universidad de Oviedo, Oviedo, Spain; ^2^Grupo de Microbiología Traslacional, Instituto de Investigación Sanitaria del Principado de Asturias (ISPA), Oviedo, Spain; ^3^Laboratorio de Referencia de Escherichia coli (LREC), Departamento de Microbioloxía e Parasitoloxía, Facultade de Veterinaria, Universidade de Santiago de Compostela (USC), Lugo, Spain; ^4^Servicio de Microbiología, Hospital Universitario Central de Asturias (HUCA), Oviedo, Spain; ^5^Laboratorio de Salud Pública (LSP), Consejería de Sanidad del Principado de Asturias, Oviedo, Spain; ^6^Plataforma de Genómica y Bioinformática, Centro de Investigación Biomédica de La Rioja (CIBIR), Logroño, Spain; ^7^Instituto de Productos Lácteos de Asturias, Consejo Superior de Investigaciones Científicas (IPLA-CSIC), Villaviciosa, Spain; ^8^Grupo de Microbiología Molecular, Instituto de Investigación Sanitaria del Principado de Asturias (ISPA), Oviedo, Spain; ^9^Departamento de Bioquímica y Biología Molecular, Universidad de Oviedo, Oviedo, Spain

**Keywords:** colistin resistance, *mcr-1*, *bla*_CMY–2_, IncX4, IncH12, IncI2, food – borne pathogens, European ST34 monophasic clone

## Abstract

Colistin is a last-resort antibiotic in fighting severe infections caused by multidrug resistant Gram negative pathogens in hospitals. Zoonotic bacteria acquire colistin resistance in animal reservoirs and mediate its spread along the food chain. This is the case of non-typhoid serovars of *Salmonella enterica*. Colistin-resistant *S. enterica* in foods represents a threat to human health. Here, we assessed the prevalence of colistin-resistance in food-borne isolates of *S. enterica* (2014–2019; Asturias, Spain), and established the genetic basis and transferability of this resistance. Five out of 231 isolates tested (2.2%) were resistant to colistin. Four of them, belonging to the European monophasic ST34 clone of *S*. Typhimurium, were characterized in the present study. They were collected from pork or pork and beef meat-derived products, either in 2015 (three isolates) or 2019 (one isolate). Molecular typing with XbaI-PFGE and plasmid profiling revealed distinct patterns for each isolate, even though two of the 2015 isolates derived from the same sample. The MICs of colistin ranged from 8 to 16 mg/L. All isolates carried the *mcr-1.1* gene located on conjugative plasmids of the incompatibility groups IncX4 (2015 isolates) or IncHI2 (2019 isolate). Apart from colistin resistance, the four isolates carried chromosomal genes conferring resistance to ampicillin, streptomycin, sulfonamides and tetracycline [*bla*_TEM–1_, *strA-strB*, *sul2*, and *tet*(B)] and heavy metals, including copper and silver (*silESRCFBAGP* and *pcoGE1ABCDRSE2*), arsenic (*arsRSD2A2BCA1D1*) ± mercury (*merEDACPTR*), which are characteristically associated with the European ST34 monophasic clone. The 2019 isolate was also resistant to other antibiotics, comprising third generation cephalosporins and cephamycins. The latter phenotype was conferred by the *bla*_CMY–2_ gene located on an IncI1-I(α)-ST2 plasmid. Results in the present study identified meat-derived products as a reservoir of a highly successful clone harboring transferable plasmids which confer resistance to colistin and other clinically important antibiotics. An important reduction in the number of food-borne *S. enterica* detected during the period of the study, together with the low frequency of colistin resistance, underlines the success of One Health initiatives, such as those implemented at the UE, to control zoonotic bacteria along the food chain and to halt the spread of antimicrobial resistance.

## Introduction

Non-typhoidal serovars of *Salmonella enterica* subsp. *enterica* are one of the leading causes of bacterial food-borne infections in humans and animals worldwide (Majowicz et al., 2010). Up to date, more than 1,500 non-typhoidal serovars of this subsp. have been recognized (Grimont and Weill, 2007; Issenhuth-Jeanjean et al., 2014), with *S*. Typhimurium being one of the most frequently detected. Since around 2005, a monophasic variant of this serovar with the antigenic formula 4,[5],12:i:- and sequence type (ST) 34 has emerged in Europe, originally in pigs and later in other domestic animals (EFSA, 2010; Hauser et al., 2010; Hopkins et al., 2010; Sun et al., 2020). Subsequently, this “European monophasic clone” has experienced a global expansion, being also responsible for multiple outbreaks and sporadic cases of human salmonellosis in America, Asia, and Australia (Soyer et al., 2009; Mulvey et al., 2011, 2013; Arai et al., 2018; Elnekave et al., 2018; Sun et al., 2020).

The European clone harbors two chromosomal genetic elements, namely the RR region and SGI-4, both involved in resistance. Isolates of this clone are typically resistant to ampicillin, streptomycin, sulfonamides and tetracycline (tetra-resistance pattern ASSuT) with the responsible genes, *bla*_TEM–1_, *strA*-*strB*, *sul2*, and *tet*(B), located on RR that also contains a mercury resistance locus. This region consists of one or two resistance modules flanked and interspersed with IS*26* elements (Lucarelli et al., 2012; Boland et al., 2015; García et al., 2016). In most cases, the tetra-R region is located upstream of *iroB*, replacing a number of chromosomal ORFs including the *fljAB* genes responsible for expression of the second flagellar phase. In addition to the tetra-R region, the European clone contains genomic island SGI-4, formerly known as SGI-3, an integrative conjugative element (ICE), which includes a copper homeostasis and silver resistance island (CHASRI), and an arsenic resistance locus (Petrovska et al., 2016; Arai et al., 2018; Branchu et al., 2019; Mourao et al., 2020). It has been proposed that the use of heavy metals as growth promoters in pork production, particularly after the ban of antibiotics with such an aim in European Union (EU), could have contributed to the epidemiological success of the clone (Mastrorilli et al., 2018; Clark et al., 2020).

Apart from chromosomal resistance to traditional antibiotics and heavy metals, the European monophasic clone is actively evolving mainly through acquisition of plasmids which can confer resistance to additional antimicrobial agents, including those categorized by the World Health Organization (WHO) as critically important with highest priority in human medicine (WHO, 2019). To this category belongs colistin (polymyxin E), a last-resort antibiotic used to treat life-threatening infections caused by multidrug-resistant, carbapenemase-producing Gram-negative bacteria in hospitals (Kaye et al., 2016). In consequence, the emergence and rapid spread of plasmid-borne colistin resistance, encoded by *mcr* genes, is a cause of serious concern in human medicine, and is actively monitored as part of programs implemented by both national and international organizations (Hugas and Beloeil, 2014). Zoonotic bacteria, like *S. enterica*, which have acquired plasmid-mediated colistin resistance, can be transmitted to humans across the food chain. Once in humans, apart from causing disease, they may act as a vehicle for the spread of colistin resistance to other *Enterobacterales*, including important nosocomial pathogens, such as *Klebsiella pneumoniae*, *Escherichia coli* and *Enterobacter* spp., and to members of the indigenous microbiota.

In the present study, experimentally obtained information was combined with whole genome sequence analysis for in-depth characterization of four food-borne monophasic isolates of *S*. Typhimurium, selected on the basis of colistin resistance, and detected in the frame of the Spanish contribution to the monitoring and reporting of antimicrobial resistance in zoonotic and commensal bacteria (Commission implementing decision 2013/652/EU; European Commission, 2013). One of them was also resistant to many other antibiotics, comprising third generation cephalosporins and cephamycins, aggravating the challenge to food-safety and public health.

## Materials and Methods

### Bacterial Isolates, Antimicrobial Susceptibility Testing and Screening of *mcr* and *bla*_CMY–2_ Genes

All *S. enterica* isolates (*n* = 231) detected in retail food samples by the Laboratory of Public Health (LSP) of the Principality of Asturias, Spain, along the 2014 to 2019 period, were tested for colistin susceptibility. MICs with Sensititre were determined for isolates recovered during 2014, 2015, 2016, and 2019, using the EUVSEC panel (for susceptibility testing of *Salmonella* and *Escherichia coli* as part of surveillance programs; Thermo Scientific). The panel consisted of ampicillin, cefotaxime, ceftazidime, meropenem, gentamicin, tetracycline, tigecycline, chloramphenicol, azithromycin, sulfamethoxazole, trimethoprim, nalidixic acid, ciprofloxacin, and colistin. For the 2017 and 2018 isolates, screening of colistin resistance was performed on SuperPolymyxin medium (Nordmann et al., 2016). Colistin MICs of resistant isolates were accurately determined by broth microdilution, according to current EUCAST recommendations^1^. The presence of *mcr-1* to *mcr-5* genes was screened by PCR using previously reported primers and conditions (Liu et al., 2016; Xavier et al., 2016; Borowiak et al., 2017; Carattoli et al., 2017; Yin et al., 2017). Disk diffusion assays were done for all monophasic *S*. Typhimurium isolates (*n* = 84) detected during the period of study. The following commercially available disks (Thermo Scientific), with the amount in μg shown in parenthesis, were used: ampicillin (10), amoxicillin-clavulanic acid (30), cefepime (30), cefotaxime (30), cefoxitin (30), ertapenem (10), chloramphenicol (30), amikacin (30), gentamicin (10), kanamycin (30), streptomycin (10), tobramycin (10), azithromycin (15), nalidixic acid (30), ciprofloxacin (5), sulfonamides (300), tetracycline (30), trimethoprim (5), fosfomycin (300), and nitrofurantoin (300). Results were interpreted according to EUCAST (2016) or CLSI (2019). For a single colistin-resistant isolate that was also resistant to both cefotaxime and cefoxitin, the presence of the *bla*_CMY–2_ gene was tested by PCR, using the primers reported by Pérez-Pérez and Hanson (2002).

To place the colistin-resistant isolates within context, information regarding year of recovery, sample of origin, serotype, as well as phage type and antimicrobial resistance properties, when known, is shown in Supplementary Table 1 for the 231 food-borne isolates. They were recovered from retail food of different origins, including fresh or processed meat from beef, pig, wild boar and chicken, eggs and derived products, seafood, and also bovine and pig carcasses at slaughterhouses.

### Experimental Typing of the Isolates and Plasmid Analysis

The serotype of all isolates, and also the phage type of those recovered before 2017, were determined by the “Agencia Española de Consumo, Seguridad Alimentaria y Nutrición” (AECOSAN). The phage type of the 2019 isolate is unknown, because since 2017 reference laboratories in Spain have discontinued typing of *Salmonella* with this technique. Typing by Pulsed-Field Gel Electrophoresis (PFGE) was done with the XbaI endonuclease (Thermo Fisher Scientific), following the PulseNet protocol for *S. enterica*^2^. XbaI-digested DNA of *S*. Braenderup H9812 was used as size standard (Hunter et al., 2005), and a dendrogram of similarity was constructed with Bionumerics v.6.6 (Applied Maths N.V., Sint-Martens-Latem, Belgium), using the unweighted pair group method with arithmetic means (UPGMA). For plasmid analysis, a modified alkaline lysis method and the S1-PFGE technique were applied (Kado and Liu, 1981; Barton et al., 1995). Plasmids of the *E. coli* strains V517 (Sanchez et al., 1986) and 39R861 (Threlfall et al., 1986) were included as size standards for undigested DNA. Conjugation experiments were performed in Luria-Bertani (LB) liquid medium, using each of colistin-resistant isolates as donors and *E. coli* K-12 J53 (resistant to rifampicin) as the recipient. Cultures of donor and recipient strains were incubated overnight at 37^°^C. Then 100 μl of the donor culture and 200 μl of the recipient culture were added to 1 ml LB. The mixtures were incubated overnight without shaking either at 37^°^C (LSP 237/15, LSP 295/15, and LSP 298/15) or at 28 and 37^°^C (LSP 38/19). Transconjugants were selected on eosin–methylene blue agar (Oxoid, Madrid, Spain), containing rifampicin (50 mg/L) plus either colistin (3.5 mg/L; for all isolates) or cefotaxime (8 mg/L; for LSP 38/19). Eight to twelve transconjugants per mating experiment were tested for antimicrobial susceptibility, plasmid content, PBRT of the IncX4, IncHI2, IncI1 and IncFIB/IncFIC replicons (Carattoli et al., 2005), and PCR detection of the *mcr-1.1* and *bla*_CMY–2_ genes. The frequencies of plasmid transfer were calculated as the number of transconjugants per donor cell, with values corresponding to the average of three independent experiments.

### Whole Genome Sequencing of the Colistin Resistant Isolates and Bioinformatic Analysis

WGS of colistin resistant isolates was performed with Illumina. Total DNA was extracted from overnight cultures grown in LB broth using the GenElute™ Bacterial Genomic DNA Kit (Sigma) according to the manufacturer’s instructions. Paired-end reads of 150 nt were generated from ca. 500–400 bp fragment libraries at the CIBIR (Centro de Investigación Biomédica, La Rioja, Spain; 2015 isolates) or at Eurofins Genomics (Ebersberg, Germany; 2019 isolate), using HiSeq 2500 and NovaSeq 6000 S2 PE150 XP platforms, respectively. Genome reconstruction was achieved with PLACNETw^3^. This tool facilitates full genome analysis, including *de novo* assembly of all the genome with VelvetOptimizer, followed by the separation of contigs belonging to the chromosome or contigs belonging to Mobile Genetic Elements (MGEs), such as plasmids. At the same time, and in a friendly, easy, and graphical way, PLACNETw allows the exploration of close references to each assembled contig, in order to determine its genetic nature (chromosome, plasmid, ICE, IME, etc.). Besides, this tool can ease MGE typing, both by the analysis of replication initiation proteins and relaxases, which are key elements in plasmid replication and mobilization. Finally, an *in silico* incompatibility group classification is performed (Lanza et al., 2014; Vielva et al., 2017).

Information regarding the quality of the assemblies, as provided by VelvetOptimiser, is compiled in Supplementary Table 2. The genomes were deposited in GenBank, under the accession numbers indicated below, and annotated by the NCBI Prokaryotic Genome Annotation Pipeline (PGAP^4^). Bioinformatic analysis was performed both with PLACNET and with the MLST, ResFinder, PlasmidFinder and pMLST tools of the Center for Genomic Epidemiology^5^ (Camacho et al., 2009; Larsen et al., 2012; Carattoli et al., 2014; Bortolaia et al., 2020; Zankari et al., 2020). SGI-4, the tetra-R and *fljAB*-*hin* regions, as well as relevant plasmids or plasmid contigs were manually refined by means of BLASTn, CLONE Manager (CloneSuite9), and MyDbFinder (CGE, DTU) using customized databases comprising open reading frames from strains SLK-521 (SGI-4; accession number MN730128), 105/7/03 and 07-2006 (tetra-R regions; accession numbers HQ331538 and KR856283, respectively), and LT2 (*fljAB-hin* region; accession number NC_003197), with STM ORFs following the *S*. Typhimurium LT2 nomenclature. Reconstruction of the IncX4 plasmids from contigs identified with PLACNET, and of the tetra-R regions (guided by the location and orientation of the multiple IS*26* elements carried by them), was achieved by PCR amplifications of the intervening DNA, followed by Sanger sequencing of the obtained amplicons (performed at STAB VIDA, Caparica, Portugal). Once established, the organization of the tetra-R regions, as well as the genetic environments of the *mcr-1.1* and *bla*_CMY–2_ genes, were represented with EasyFig^6^.

### Nucleotide Sequence Accession Numbers

The genome sequences of the colistin resistant isolates under study were deposited at the GenBank database with the following accession numbers: JACXKV0000000 00, JACXKU000000000, JACXKT000000000, and JAGMWH00 0000000, for LSP 237/15, LSP 295/15, LSP 298/15, and LSP 38/19, respectively. The plasmid sequences of pIncX4_LSP 237/15, pIncX4_LSP 295/15, pIncX4_LSP 298/15, pIncHI2_contig 309_LSP 38/19, and pIncI1-I(α)_contig 9_LSP 38/19 were also deposited in GenBank under accession numbers OK642377, OK642378, OK642379, OK642375, and OK642376.

## Results

### General Characteristics of the Isolates, Typing and Plasmid Analysis

During the 2014 to 2019 period, 231 isolates of *S. enterica* were recovered from food samples in Asturias. Five of them (2.2%) were resistant to colistin and four of these (LSP 237/15, LSP 295/15 and LSP 298/15 and LSP 38/19), pertaining to the monophasic European clone of *S*. Typhimurium were thoroughly characterized in the present study (Table 1). They represent 4.8% of the total number of isolates belonging to this clone (84; 36.4% of the total *S. enterica*) detected in food samples during the period of study. The remaining isolate, LSP 136/15, was identified as *S. enterica* serovar Kedougou and carried the *mcr-4.3* gene on a ColE10 plasmid (Rebelo et al., 2018).

**TABLE 1:** Origin and properties of food-borne colistin-resistant isolates of the monophasic ST34 variant of *Salmonella enterica* serovar Typhimurium.

Isolate^a^	Origin	Antigenic formula	Phage type^b^	Colistin MIC (mg/L)	Resistance phenotype (capitalized)^c^ Antibiotic resistance genes (italicized)^c^	Plasmid (size in bp)^d^
LSP 237/15	Pork and beef meat processed for raw consumption	4,12:i:-	PT 104B	16	COL, AMP, STR, SUL, TET *mcr-1.1*, *bla*_TEM–1_, [*strA*, *strB*], *sul2*, *tet*(B)	IncI2(δ) (61,356) **IncX4** (33,336)* ColE1-like (5,055)* ColE1-like (3,351)*
LSP 295/15	Pork and beef minced meat	4,5,12:i:-	PT 138	8	COL, AMP, STR, SUL, TET *mcr-1.1*, *bla*_TEM–1_, [*strA*, *strB*], *sul2*, *tet*(B)	**IncX4** (33,304)*
LSP 298/15	Pork and beef minced meat	4,5,12:i:-	RDNC	8	COL, AMP, STR, SUL, TET *mcr-1.1*, *bla*_TEM–1_, [*strA*, *strB*], *sul2*, *tet*(B)	IncI1-I(α)-ST_unk_ (88,771) **IncX4** (33,304)*
LSP 38/19	Fresh pork sausage	4,5,12:i:-	ND	8	COL, AMP, [CTX, FOX], CHL, [STR, GEN, TOB, KAN], SUL, TET, TMP *mcr-1.1, bla_*TEM–1*_, bla*_CMY–2_, [*cmlA1, floR*], [*strA*, *strB*, *aadA1, aadA2*, *aac(3)-IV, aph(3′)-Ia, aph(4)-Ia*], *sul2*, *sul3*, [*tet*(A), *tet*(B), *tetM*], *dfrA12*	**IncHI2** (242,363) **IncI1-I(α)-ST2** (105,151) **IncFIB, IncFIC-F46:A-:B20** (84,823) **ColE1** (5,699)* ColE1 (4,664)* UNK (3,751)*

*^a^LSP, “Laboratorio de Salud Pública, Principado de Asturias,” Spain. The names of the isolates include a serial number followed by the last two digits of the year of isolation. LSP 295/15 and LSP 298/15 originated from the same sample.*

*^b^PT, phage type; RDNC, reacted but did not conform; ND, not determined.*

*^c^COL, colistin; AMP, ampicillin; CTX, cefotaxime; FOX, cefoxitin; CHL, chloramphenicol; STR, streptomycin; GEN, gentamicin; TOB, tobramicin; KAN, kanamycin; SUL, sulfonamides; TET, tetracycline; TMP, trimethoprim. Resistance to hygromycin, encoded by the aph(4)-1a gene, was not experimentally tested. Antibiotics pertaining to the same family, as well as genes conferring resistance to the same family of antibiotics are enclosed in square brackets.*

*^d^Inc, plasmid incompatibility group; UNK, unknown. IncX4 (mcr-1.1), IncHI2 (mcr-1.1 and other resistance genes) and IncI1-I(α) (bla_CMY–2_), as well as two other resistance plasmids, IncFIB, IncFIC, and ColE1, are highlighted in bold (see text for details). Circularized plasmids are marked with an asterisk.*

The selected isolates were recovered from retail meat products either in 2015 (LSP 237/15, LSP 295/15, and LSP 298/15) or 2019 (LSP 38/19). Remarkably, although LSP 295/15 and LSP 298/15 derived from the same sample (pork and beef minced meat), the four isolates were different (see below), and could be then assigned to distinct strains. As shown in Table 1, the antigenic formula of the colistin resistant isolates was 4,12:i:- (LSP 237/15) or 4,5,12:i:- (LSP 295/15, LSP 298/15, and LSP 38/19). The three isolates from 2015 belonged to phage types DT104B, DT138 and RDNC, while the phage type of the 2019 isolate was not determined.

**FIGURE 1:**
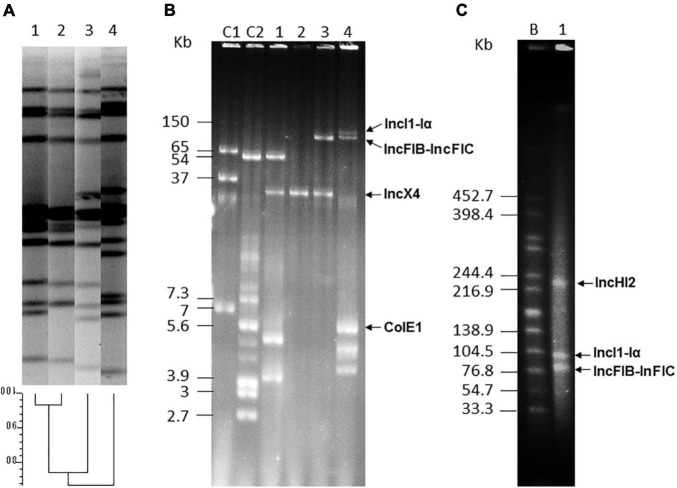
Typing of food-borne isolates belonging to the European monophasic clone, and plasmid analysis. **(A)** XbaI-PFGE and dendrogram showing the similarity between the generated profiles. Lane 1, LSP 295/15; lane 2, LSP 298/15; lane 3, LSP 38/19; lane 4, LSP 237/15. **(B)** Plasmid profiles generated with the Kado-Liu method. Lanes C1 and C2, plasmids obtained from *Escherichia coli* 39R861 (NCTC 50192) and V517 (NCTC 50193) used as size standards for undigested DNA; lane 1, LSP 237/15; lane 2, LSP 295/15; lane 3, LSP 298/15; lane 4, LSP 38/19. **(C)** S1-PFGE. Lane B, XbaI-digested DNA of *Salmonella enterica* serovar Braenderup H9812 used as size standard; lane 1, LSP 38/19. Resistance plasmids are indicated in **(B,C)**.

Draft genomes of the isolates consisted in 79 to 218 total contigs (23 to 88 larger than 1 kb), with assembly sizes ranging from 4.94 to 5.42 Mb (Supplementary Table 2). MLST typing performed “*in silico*” assigned the isolates to ST34, as expected for the European monophasic clone. Experimental typing with XbaI-PFGE revealed a distinct pattern for each isolate, though profiles of the two isolates obtained from the same sample (LSP 295/15 and LSP 298/15) were more closely related (Figure 1A). Plasmids were found in all isolates, in numbers ranging from one (LSP 295/15) up to six (LSP 38/19). They belonged to different incompatibility groups and each isolate, even those derived from the same sample, had a different plasmid profile (Figures 1B,C; Table 1).

### Resistance Properties and Genetic Basis of Antimicrobial Drug Resistance

As shown in Table 1, MICs of colistin for the isolates ranged from 8 to 16 mg/L, and the *mcr-1.1* gene was identified in all of them. The gene was carried by IncX4/MOBP3 plasmids of 33.3 kb in the 2015 isolates, and by a large IncHI2/MOBH plasmid of 242 kb in the 2019 isolate (Figures 1B,C; Table 1). Chromosomal mutations in the *pmrA/B* regulatory genes, which can also mediate colistin resistance by affecting the expression of enzymes involved in modification of the lipopolysaccharide, were not observed. Apart from colistin resistance, the isolates shared the tetra-resistance pattern (ampicillin, streptomycin, sulfonamides and tetracycline), characteristically associated with the monophasic ST34 clone and conferred by *bla*_TEM–1_, *strA*-*strB*, *sul2*, and *tet*(B) genes of chromosomal location. Moreover, LSP 38/19 was also resistant to cefotaxime and cefoxitin. This phenotype is consistent with production of an AmpC β-lactamase, and the presence of *bla*_CMY–2_ was indeed demonstrated. The gene, which encodes the CMY-2 AmpC β-lactamase, resided on a IncI1-I(α)/*mckB*/MOBP plasmid, assigned to ST2 by pMLST. The 2019 isolate was additionally resistant to many other antibiotics, including chloramphenicol, aminoglycosides (gentamicin, kanamycin, and tobramycin), and trimethoprim, with the responsible genes located on the large IncHI2 [*aadA1*, *aadA2*, *aac(3 ″)-IId*, *cmlA1*, *floR*, *dfrA12*, and *tetM*] and IncFIB-IncFIC/*traI*/MOBH [*aadA1*, *cmlA1*, *sul3, tet*(A), and *merRTPC*] plasmids, or carried by a small ColE1 plasmid [*aph(3′)-Ia*]. The actual location of two additional aminoglycoside resistance genes, *aph(4)-1a* and *aph(3)-IV*, which were found together, flanked by two copies of IS26, could not be established.

### Genetic Environment and Transferability of the *mcr-1.1* and *bla*_CMY–2_ Genes

**FIGURE 2:**
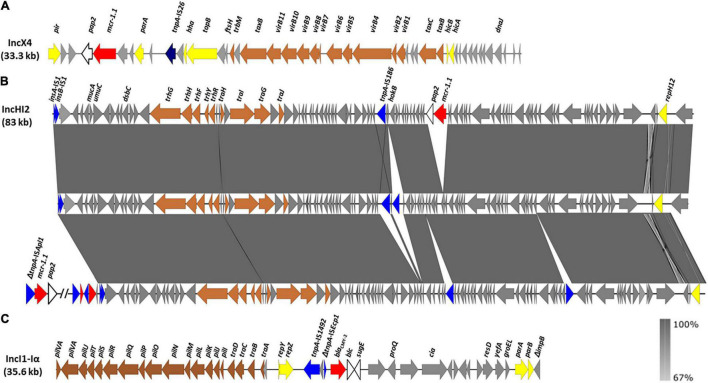
Genetic environment of the *mcr-1.1* and *bla*_CMY–2_ genes detected in food-borne isolates of the European monophasic clone. **(A)** Genetic context of *mcr-1.1* carried by IncX4 plasmids in the LSP 237/15, LSP 295/15, and LSP 298/15 isolates. **(B)** Genetic organization of a ca. 83 kb contig carrying the *mcr-1.1* gene in the IncHI2 plasmid of LSP 38/19. The alignment of this contig with homologous regions in two related plasmids either lacking *mcr-1.1* (pKUSR18; accession number KM396298) or having the gene outside this region (pSE08-00436-1; accession number NZ_CP020493) was created with EasyFig BLASTn. The gray shading between the regions reflects nucleotide sequence similarity ranging from 67 to 100%, according to the scale shown at the lower right part of the figure. **(C)** Genetic environment of the *bla*_CMY–2_ gene in a 35.6 kb contig of the IncI1-I(α) plasmid of LSP 38/19. The open reading frames (ORFs) are represented by arrows pointing to the direction of transcription and having different colors according to their function: red, resistance; yellow, plasmid replication, stability and maintenance; brown, conjugation; blue, DNA mobility, with the *tnpA* gene of IS*26* shown in dark blue; gray, other roles; white, *pap2*, adjacent to *mcr-1.1* in the IncX4 and IncHI2 plasmids, and *bcl*-*sugE*, adjacent to *bla*_CMY–2_ in the IncI1-I(α) plasmid. The scale is different for **(A–C)**.

The *mcr-1.1* gene was located on IncX4 plasmids in the 2015 isolates, and carried by a large IncHI2 plasmid in the 2019 isolate (Table 1). The genetic context of *mcr-1.1* in the 2015 isolates is shown in Figure 2A. As previously reported for other IncX4 plasmids, *mcr-1.1* was found adjacent to a putative *orf*, designated *pap2* since it encodes a transmembrane protein of the PAP2 superfamily. The IS*Apl1* insertion sequence, proposed to be involved in the initial mobilization of *mcr-1*, likely by means of the Tn*6330* composite transposon (see below), is not detected (Li et al., 2017). However, a single copy of IS*26*, flanked by 8 bp target site duplications (CTGTGTGA), is located further downstream. The IncX4 plasmids of LSP 295/15 and LSP 298/15 were identical to each other, and differed from the IncX4 plasmid of LSP 237/15 by a single SNP and a 32 bp insertion present in the latter one but not in the former two. The IncX4 plasmids of these isolates were very closely related (more than 99% identity and coverage) to previously sequenced IncX4 plasmids carrying either *mcr-1.1* (such as pE15004 from *E. coli*; accession number KX772777), or *mcr-1.2* (like pMCR1.2-IT from *K. pneumoniae*; accession number KX236309).

The *mcr-1.1* gene of the 2019 isolate was located on a large contig of 83 kb (Figure 2B), which belongs to the IncHI2/MOBH plasmid. As in the 2015 isolates, the gene was adjacent to *pap2* and devoided of IS*AplI*. The insertion site of *mcr-1.1*-*pap2*, between two *orfs* encoding hypothetical proteins, was previously found only in unnamed plasmid1 from *Salmonella* isolate S15BD05371 which, according to SeqSero and MLST, corresponds to the monophasic ST34 variant of *S*. Typhimurium (accession number GCF_014857405.1). Other IncH12 plasmids contain regions closely related to the 83 kb contig of the LSP 38/19 plasmid, in which the *mcr-1.1* gene is either absent (i.e., pKUSR18; accession number KM396298) or placed outside the corresponding region (i.e., pSE08-00436-1; accession number NZ_CP020493) (Figure 2B). On the other hand, the *bla*_CMY–2_ gene of LSP 38/19 resided on an IncI1-I(α)-ST2 plasmid of 105 kb (Table 1). More precisely, *bla*_CMY–2_ was located on a contig of 35.6 kb, which harbored the IS*Sbo1*-like-ΔIS*Ecp1*-*bla*_CMY–2_-*blc*-*sugE* structure, inserted downstream of the *repZ* gene (Figure 2C). This contig was more closely related to regions of IncI1-I(α)-ST2 plasmids found in *E. coli* isolates from a human patient (pC-6; accession number KT186369) and a dog (pR7AC; accession number KF434766).

The IncX4, IncHI2 and IncI1-I(α) plasmids harboring the *mcr-1.1* or *bla*_CMY–2_ genes in the LSP isolates could be transferred into *E. coli* through conjugation/mobilization experiments (Table 2). The frequency of conjugation of the IncX4 found alone in the LSP 295/15 isolate was of 2.6 × 10^–6^ transconjugants/donor cell. Similar frequencies were obtained for the IncX4 plasmids of LSP 237/15 and LSP 298/15, which each co-resided with a different IncI1 plasmid (Table 1), both cryptic. Taking into account that the IncX4 plasmids of the three LSP isolates were nearly identical (see above), the IncX4 plasmids of LSP 237/15 and LSP 298/15 are expected to be conjugative and, in fact, they were found alone in ca. 10% of the transconjugants analyzed. However, mobilization cannot be excluded as most transconjugants carried the IncX4 and IncI plasmids. In the case of LSP 38/19, conjugation experiments were carried out both at 28 and 37^°^C, since it has been reported that optimal transfer of IncHI2 plasmid occurs between 22 and 30^°^C (García-Fernández and Carattoli, 2010). Selection was performed either with cefotaxime or colistin, used as markers of the IncI1-I(α) and IncHI2 plasmids, respectively. At 37^°^C, selection with cefotaxime yielded a transfer frequency of 3.6 × 10^–5^ transconjugants/donor cell, while at 28^°^C transconjugants were not obtained (frequency <1.9 × 10^–8^). These results indicate that the IncI1-I(α) of LSP 38/19 can be efficiently transferred at 37^°^C but not at 28^°^C. All transconjugants analyzed from the 37^°^C mating were resistant to cefotaxime and colistin, PCR-positive for *bla*_CMY–2_ and *mcr-1.1*, and carried the IncHI2, IncI1-I(α) and IncFIB-IncFIC plasmids. In contrast, when selection was performed with colistin, the conjugation frequencies were of 2.6 × 10^–5^ and 1.6 × 10^–5^ at both 28 and 37^°^C. Transconjugants resulting from matings at the two temperatures were resistant to colistin but susceptible to cefotaxime, PCR positive for *mcr-1.1* but not for *bla*_CMY–2_, and carried the IncHI2 and IncFIB-IncFIC plasmids. These results are consistent with efficient transfer of the IncHI2 plasmid at 28^°^C, either by conjugation and/or mobilization, and also at 37^°^C, although in the latter case it has to be mobilized by the accompanying IncFIB-IncFIC plasmid.

**TABLE 2:** Conjugation/mobilization frequencies of IncX4, IncHI2, and IncI1-I(α) plasmids of food-borne colistin resistant isolates of the monophasic ST34 variant of *Salmonella enterica* serovar Typhimurium.

Donor strain / plasmid(s)	Selection for:	Selection with^a^:	Transfer frequency^b^ (standard error)	
			28^°^C	37^°^C
LSP 237/15 / IncX4; IncI2(δ)^c^	IncX4	RIF + COL	nd	**1.5 × 10**^–^**^6^** (0.98)
LSP 295/15 / IncX4	IncX4	RIF + COL	nd	**2.6 × 10**^–^**^6^** (0.85)
LSP 298/15 / IncX4; IncI1-I(α)c	IncX4	RIF + COL	nd	**0.6 × 10**^–^**^6^** (0.42)
LSP 38/19 / IncHI2; IncI1-I(α)	IncH12	RIF + COL	**2.6 × 10**^–^**^5^** (1.2)	**1.6 × 10**^–^**^5^** (1.2)
LSP 38/19 / IncHI2; IncI1-I(α)	IncI1-I(α)	RIF + CTX	<**1.9 × 10**^–^**^8^**	**3.6 × 10**^–^**^5^** (1.8)

*^a^RIF, rifampicin; COL, colistin; CTX, cefotaxime.*

*^b^Conjugation frequencies (shown in bold) are reported as the number of transconjugants per donor cell, and result from the average of three independent experiments, nd, not determined.*

*^c^Criptic plasmids for which selection is not available.*

### Chromosomal Regions Involved in Antibiotic Resistance and Heavy Metal Tolerance Genetic Bases of the Monophasic Phenotype

**FIGURE 3:**
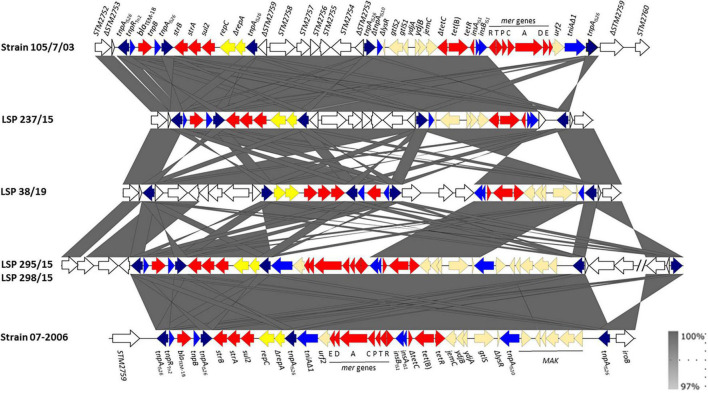
Genetic organization of the tetra-resistance regions found in food-borne isolates of the European monophasic clone. The alignment was created with EasyFig BLASTn including the tetra-resistance regions of strains 105/7/03 (accession number HQ331538, shown above) and 07-2006 (accession number KR856283; shown below) for comparison. The open reading frames (ORFs) are represented by arrows pointing to the direction of transcription and having different colors according to their origin or roles: white, chromosomal ORFs of the *S*. Typhimurium LT2 chromosome (named according to accession number NC_003197); red, resistance; blue, DNA mobility, with the *tnpA* gene of IS*26* shown in dark blue; yellow, plasmid replication genes; pale orange, other roles. The gray shading between the structures connects regions of nucleotide sequence similarity ranging from 97 to 100%, according to the scale shown at the lower right part of the figure.

As shown in Figure 3, the tetra-R regions of LSP 295/15 and LSP 298/15 were identical to each other and consisted of a contiguous module which also coincided with that of strain 07-2006, previously reported by García et al. (2016). However, the flanking DNA at the left and right borders was different (ΔSTM2756 and a large inverted segment encompassing from ΔSTM2816 to *iroB* and flanked by oppositely oriented copies of IS*26*, in the case of LSP 295/15 and LSP 298/15, and STM2759 and *iroB* in strain 07-2006). In contrast, the tetra-R regions of LSP 237/15 and LSP 38/19 comprised two modules embedded between ΔSTM2753 and *iroB*. The modules of LSP 237/15 resemble those of 105/7/03 (Lucarelli et al., 2012), except for the *mer* locus which is present in the latter but not in the former. In both cases, ΔSTM2759 to ΔSTM2753, inverted with respect to their orientation in the chromosome of *S*. Typhimurium LT2, are located between the two modules. The organization of LSP 38/19 is similar to that of LSP 237/15, except that the first module and adjacent ORFs (ΔSTM2759 to ΔSTM2753) are inverted in 38/19, and additional ORFs (ΔSTM2759 to ΔSTM2761, preceded by an IS*26* element), are found between the two modules. In all strains except 105/7/03, evolution of the tetra-R region was accompanied by deletions of a number of chromosomal ORFs that always included the *fljAB* genes, as well as the entire (LSP 295/15, LSP 298/15, LSP 38/19, and 07-2006) or part (LSP 237/15) of the *hin* gene, linking these regions with the monophasic phenotype.

In contrast to the diversity of the tetra-R regions, SGI-4 was identical in the analyzed isolates, and identical or nearly identical to the genomic islands carried by many other ST34 monophasic strains, including SLK-521 used for comparison (Mourao et al., 2020). It comprised the *silESRCFBAGP*, *pcoGE1ABCDRSE2*, and *arsRSD2A2BCA1D1* gene clusters, accountable for copper, silver and arsenic tolerance. The insertion site of the ICE element, between STM4320 and *yjdC* (both encoding putative transcriptional regulators), also coincides with previous information.

## Discussion

During the 2014 to 2019 six year period, 231 isolates of *S. enterica* were recovered from food samples in Asturias, a Northern Spanish region. Interestingly, the number of isolates decreased considerably along time, from nearly 96 in 2014 down to 4–10 since 2017, a favorable trend that probably correlates with implementation in Spain of the integrated EU legislation aimed to monitor and control *Salmonella* along the food chain (European Commission, 2005; Hugas and Beloeil, 2014). Although with some delay, this trend roughly coincides with that reported in the EU for human salmonellosis, which has been stable over the past 5 years after a long period of decline (EFSA and ECDC, 2021). In Asturias, 36.4% of the isolates recovered from foods belonged to the European monophasic clone of *S*. Typhimurium, with sequence type ST34. The incidence of this clone increased considerably along the last years, and four out of five colistin-resistant isolates detected during the period of study belonged to this clone. The ST34 isolates characterized in the present study carried *mcr-1.1* on transferable IncX4 or IncHI2 plasmids. These plasmid types are amongst the most prevalent vehicles leading to the worldwide spread of *mcr-1* in different bacteria, including the European monophasic clone (Nang et al., 2019).

There is strong evidence supporting that the *mcr-1* gene was originally mobilized from the chromosome of a species of the genus *Moraxella* by an IS*Apl1*-flanked composite transposon termed Tn*6330* (Poirel et al., 2017; Snesrud et al., 2018). However, most of the detected *mcr-1* structures lack either one (single-ended derivatives) or more frequently both copies of IS*Apl1* (Snesrud et al., 2016, 2018). This is regarded as a mechanism of stabilization of the *mcr-1* gene, associated with the loss of the transposition capacity. Neither of the two copies of IS*Apl1* were found surrounding the *mcr-1.1* genes of the isolates in the present study, although a remnant of the downstream IS*Apl1* inverted right repeat (IRR) of 27 bp, which encompasses the last 26 bp of the *pap2* gene, was identified in the IncX4 plasmids of the 2015 isolates, and a shorter version with only 21 bp, overlaps the *pap2* gene in the IncHI2 plasmid. In single-ended variants, a unique upstream copy of IS*Apl1* in conjunction with the IRR of the ancestral downstream IS*Apl1* may still be able to mobilize the *mcr-1* gene. In contrast, variants entirely devoided of IS*Apl1*, like those reported in the present study, have made their way toward stabilization of the resistance gene and maintenance of plasmid integrity (Snesrud et al., 2018). Interestingly, a copy of IS*26* is present in the IncX4 plasmid, close to the *mcr-1.1* gene. In contrast to IS*Apl1*, IS*26*, once gained by a plasmid or the chromosome, is able to proliferate, actively contributing to the generation of complex resistance regions containing multiple copies of the element (Harmer and Hall, 2014; He et al., 2015; Vazquez et al., 2021). It would be worth to follow the activity of IS*26* within IncX4 plasmids carrying *mcr-1.1*. Indeed, IS*26* appears to have played a critical role in the generation of multiple variants of the tetra-R region of the European monophasic clone. It has been proposed that a copy of IS*26*, inserted at exactly the same position adjacent to *iroB*, could have been responsible for the monophasic phenotype of this clone, by causing different deletions affecting the *fljAB* operon. The originally acquired copy of IS*26* could have then acted as a target or recognition site for incorporation of one or more antibiotic resistance modules linked to additional IS*26* elements (García et al., 2016). Such events were associated to further deletions and/or inversions of the chromosomal DNA upstream of *iroB*, yielding multiple variants of the tetra-R region, like those reported in the present study.

Interestingly, although the four isolates characterized in the present study derived from three food samples, each was assigned to a distinct strain, according to differences in phage type, XbaI-PFGE profiles, variations affecting both the tetra-R regions and the deletions responsible of the monophasic phenotype, and plasmid content. The phage type was only determined for the 2015 isolates that were assigned to DT104B, DT138 and to RDNC. Although DT193 and DT120 are the main types associated with the ST34 clone, several others types, including DT104B and DT138, have been reported (EFSA, 2010; Hopkins et al., 2010; Gallati et al., 2013; Petrovska et al., 2016; Campos et al., 2019). With regard to PFGE and plasmid profiles, the two isolates obtained from the same sample, though different, were more closely related to each other than to those recovered from different samples. Interestingly, a remarkable level of short-term microevolution has accumulated during clonal expansion of monophasic ST34 (Petrovska et al., 2016; Tassinari et al., 2020; Cadel-Six et al., 2021). This led to a large amount of genomic variation, such as that observed for the isolates analyzed in the present study. Of particular interest is the evolution of the clone through acquisition of transmissible IncX4, IncHI2 and IncI1 plasmids, which can play an important role in the spread of the *mcr-1* and *bla*_CMY–2_ genes through the food chain. CMY-2 is one of the plasmid-determined AmpC-type β-lactamases, enzymes which confer resistance to a wide range of β-lactams, including oxyimino cephalosporins and cephamycins (Philippon et al., 2002). Acquisition of this gene by *S. enterica* serotypes is a matter of concern since these compounds are drugs of choice for the treatment of severe infections caused by this and other bacteria (Gilbert et al., 2021). Furthermore, this species could act as reservoir and vehicle for transmission of *mcr-1.1* and *bla*_CMY–2_ genes to other clinical important bacterial species throughout the food chain.

In summary, the study identifies meat-derived products as a reservoir of a highly successful clone harboring “epidemic” plasmids which confer resistance to colistin and other clinically important antibiotics. One Health initiatives, as those implemented by the EU, are vital for the containment of these isolates which are potentially dangerous to human health. The observed reduction in the number of food-borne *S. enterica*, together with the low frequency of resistance to the last resort colistin, underlines the success of such measures at a regional level. It would be interesting to investigate whether similar isolates to those reported herein are also circulating in hospitals of the region.

## Data Availability Statement

The datasets presented in this study can be found in online repositories. The names of the repository/repositories and accession number(s) can be found below: https://www.ncbi.nlm.nih.gov/genbank/, JACXKV000000000; https://www.ncbi.nlm.nih.gov/genbank/, JACXKU000000000; https://www.ncbi.nlm.nih.gov/genbank/, JACXKT000000000; https://www.ncbi.nlm.nih.gov/genbank/, JAGMWH000000000; https://www.ncbi.nlm.nih.gov/genbank/, OK642377; https://www.ncbi.nlm.nih.gov/genbank/, OK642378; https://www.ncbi.nlm.nih.gov/genbank/, OK642379; https://www.ncbi.nlm.nih.gov/genbank/, OK642375; https://www.ncbi.nlm.nih.gov/genbank/, OK642376.

## Author Contributions

JF, VL, RR, and MR conceptualized and designed the study. XV, VG, and MB carried out the experiments. XV, MB, MT, RR, and MR did data curation. XV, VG, RR, and MR prepared tables and figures. XV, MT, VL, RR, and MR performed WGS analyses. XV, RR, and MR drafted the manuscript. All authors contributed to analysis and interpretation of the results, reviewed the manuscript, and approved the final version.

## Conflict of Interest

The authors declare that the research was conducted in the absence of any commercial or financial relationships that could be construed as a potential conflict of interest.

## Publisher’s Note

All claims expressed in this article are solely those of the authors and do not necessarily represent those of their affiliated organizations, or those of the publisher, the editors and the reviewers. Any product that may be evaluated in this article, or claim that may be made by its manufacturer, is not guaranteed or endorsed by the publisher.

## Footnotes

1
www.eucast.org

2
https://pulsenetinternational.org/protocols/pfge/

3
https://castillo.dicom.unican.es/upload/

4
https://www.ncbi.nlm.nih.gov/genome/annotation_prok/

5
https://cge.cbs.dtu.dk/services/

6
https://mjsull.github.io/Easyfig/
