# Supplementary material for: Colistin Resistance in Monophasic Isolates of Salmonella enterica ST34 Collected From Meat-Derived Products in Spain, With or Without CMY-2 Co-production
Source: Front Microbiol. 2022 Jan 6;12:735364. doi: 10.3389/fmicb.2021.735364 (PMC8770973; doi:10.3389/fmicb.2021.735364)
Supplement: Supplementary file 1 [file Data_Sheet_1.pdf]

Supplementary Material

Table S1. Food-borne isolates of *Salmonella enterica* recovered in Asturias along the 2014 to 2019 period.

LSP[a] isolate	Source[b]	Serotype	Phage type[c]	R profle[d]
27/14	Poultry product	Infantis 6,7:r:1,5	ND	NAL,SUL,TET
28/14	Carcass surface	Typhimurium 4,12:i:1,2	U302	AMP,CHL,NAL,SMX,TET
33/14	Red dry sausage	Typhimurium 4,5,12:i:1,2	PT120	AMP,CHL,NAL,SMX,TET
34/14	Red dry sausage	Typhimurium 4,5,12:i:1,2	PT120	AMP,CHL,GEN,SMX,TET
35/14	Red dry sausage	Typhimurium 4,5,12:i:1,2	PT120	AMP,CHL,GEN,SMX,TET
36/14	Red dry sausage	Typhimurium 4,5,12:i:1,2	PT120	AMP,SMX,TET
38/14	Red dry sausage	Typhimurium 4,5,12:i:1,2	RDNC	AMP,SMX,TET
39/14	Carcass surface	Typhimurium 4,12:i:1,2	U302	AMP,CHL,NAL,SMX,TET
40/14	Carcass surface	Typhimurium 4,12:i:1,2	PT120	AMP,CHL,GEN,SMX,TET
41/14	Red dry sausage	Typhimurium 4,5,12:i:1,2	PT120	AMP,CHL,GEN,NAL,SMX,TET
42/14	Red dry sausage	Typhimurium 4,5,12:i:1,2	PT120	AMP,CHL,GEN,SMX,TET
61/14	Red dry sausage	Typhimurium 4,12:i:-	PT138	AMP,STR,SMX,TET
62/14	Red dry sausage	Typhimurium 4,12:i:-	PT138	AMP,STR,SMX,TET
63/14	Red dry sausage	Typhimurium 4,5,12:i:1,2	PT120	AMP,STR,SMX,TET
64/14	Red dry sausage	Bovismorbificans	ND	AMP,CHL,NAL,TET
65/14	Red dry sausage	Derby 4,12:f,g:-	ND	STR,SMX,TET
112/14	Red dry sausage	Typhimurium 4,12:i:1,2	PT195	AMP,CHL,NAL,TET
113/14	Carcass surface	Rissen 6,7:fg:-	ND	AMP,TET
114/14	Carcass surface	Typhimurium 4,5,12:i:-	PT193	AMP,STR,SMX,TET
115/14	Carcass surface	Rissen 6,7:fg:-	ND	AMP,CHL,TET
116/14	Carcass surface	Typhimurium 4,12:i:1,2	UT	AMP,TET
121/14	Carcass surface	Rissen 6,7:fg:-	ND	AMP,SMX,TET
122/14	Carcass surface	Rissen 6,7:fg:-	ND	AMP,TET
131/14	Carcass surface	Rissen 6,7:fg:-	ND	AMP,CHL,SMX,TET

132/14	Carcass surface	Typhimurium 4,5,12:i:1,2	PT120	AMP,CHL,SMX,TET
133/14	Carcass surface	London 3,10:I,v:1,6	ND	AMP,TET
134/14	Carcass surface	Rissen 6,7:fg:-	ND	AMP,SMX,TET
135/14	Carcass surface	Rissen 6,7:fg:-	ND	AMP,CHL,TET
136/14	Carcass surface	London 3,10:I,v:1,6	ND	AMP,CHL,NAL,SMX,TET,TMP
137/14	Carcass surface	London 3,10:I,v:1,6	ND	AMP,CHL,SMX,TET,TMP
138/14	Carcass surface	Typhimurium 4,12:i:-	PT193	AMP,STR,SMX,TET
151/14	Carcass surface	Anatum 3,10:d:1,2	ND	AMP,CHL,NAL,SMX,TET
152/14	Red dry sausage	Anatum 3,10:d:1,2	ND	AMP,CHL,NAL,SMX,TET,TMP
153/14	Red dry sausage	Anatum 3,10:d:1,2	ND	AMP,CHL,NAL,SMX,TET,TMP
154/14	Red dry sausage	Typhimurium 4,5,12:i:-	PT138	TET
159/14	Carcass surface	Typhimurium 4,12:i:1,2	UT	AMP,CHL,NAL,SMX,TET
160/14	Carcass surface	Typhimurium 4,12:i:1,2	PT195	AMP,CHL,NAL,SMX,TET,TMP
161/14	Carcass surface	Typhimurium 4,12:i:1,2	PT195	AMP,SMX,TET,TMP
162/14	Carcass surface	Typhimurium 4,12:i:1,2	UT	NAL,TET
163/14	Carcass surface	Typhimurium 4,12:i:1,2	PT195	AMP,NAL,SMX,TET,TMP
164/14	Carcass surface	Typhimurium 4,12:i:1,2	PT195	AMP,SMX,TET
165/14	Carcass surface	Typhimurium 4,12:i:1,2	PT138	AMP,STR,SMX,TET
166/14	Carcass surface	Anatum 3,10:d:1,2	ND	AMP,CHL,NAL,SMX,TET
167/14	Dry meat product	Anatum 3,10:d:1,2	ND	AMP,CHL,TET
168/14	Dry meat product	Anatum 3,10:d:1,2	ND	AMP,CHL,NAL,SMX,TET
169/14	Dry meat product	Anatum 3,10:d:1,2	ND	CHL,TET
170/14	Dry meat product	Anatum 3,10:d:1,2	ND	CHL,NAL,TET
171/14	Dry meat product	Anatum 3,10:d:1,2	ND	AMP,CHL,NAL,TET
172/14	Dry meat product	Anatum 3,10:d:1,2	ND	AMP,CHL,NAL,SMX,TET
173/14	Dry meat product	Anatum 3,10:d:1,2	ND	TET
174/14	Dry meat product	Anatum 3,10:d:1,2	ND	AMP,CHL,NAL,SMX,TET
175/14	Dry meat product	Anatum 3,10:d:1,2	ND	TET
185/14	Dry meat product	Anatum 3,10:d:1,2	ND	AMP,TET
186/14	Stewed meat	Anatum 3,10:d:1,2	ND	TET
187/14	Stewed meat	Anatum3,10:d:1,2	ND	TET

188/14	Stewed meat	Anatum3,10:d:1,2	ND	CHL,NAL,SMX,TET
221/14	Tuna fish roll	Coeln 4,5,12:y:1,2	ND	S
273/14	Clams	Napoli 9,12:l,z13:e,n,x	ND	S
274/14	Clams	Ndolo 1,9,12:d:1,5	ND	CHL
291/14	Razor clams	Typhimurium 4,5,12:i:-	RDNC	AMP,CHL,STR,SMX,TET
292/14	Fresh meat product	Typhimurium 4,12:i:-	PT193	TET
293/14	Fresh meat product	Typhimurium 4,12:i:-	PT104B	AMP,STR,SUL,TET
294/14	Fresh meat product	Typhimurium 4,5,12:i:1,2	U310	AMP,TET
295/14	Fresh meat product	Typhimurium 4,5,12:i:-	UT	STR,TET
296/14	Fresh meat product	Typhimurium 4,12:i:1,2	U302	AMP,CHL,NAL,SMX,TET
297/14	Dry creole sausage	Typhimurium 4,12:i:1,2	U302	AMP,CHL,NAL,SMX,TET
298/14	Dry creole sausage	Typhimurium 4,12:i:1,2	U302	AMP,STR,SMX,TET
299/14	Dry creole sausage	Typhimurium 4,12:i:1,2	U302	AMP,CHL,NAL,SMX,TET
300/14	Dry creole sausage	Typhimurium 4,12:i:1,2	U302	AMP,CHL,NAL,SMX,TET
301/14	Dry creole sausage	Enteritidis 1,9,12.g,m:-	ND	AMP,CHL,NAL,SMX,TET,AZM
302/14	Meat product	Amberg 6,14,24:l,v:1,7	ND	AMP,CHL,NAL,SMX,TET
305/14	Fresh meat product	Typhimurium 4,12:i:1,2	PT195	AMP,CHL,NAL,SMX,TET
306/14	Meat product	Brandenburg 1,4,12:l,v:e,n,z15	ND	AMP,CHL,NAL,SMX,TET
388/14	Meat product	Typhimurium 4,12:i:-	RDNC	AMP,STR,SMX,TET
389/14	Fresh meat product	Typhimurium 4,12:i:-	PT104B	AMP,STR,SMX,TET
390/14	Fresh meat product	Typhimurium 4,12:i:-	PT104B	AMP,STR,SMX,TET
391/14	Fresh meat product	Typhimurium 4,12:i:-	PT104B	AMP,STR,SMX,TET
393/14	Fresh meat product	Derby 4,12:f,g:-	ND	STR,SMX,TET
394/14	Meat product	Typhimurium 4,5,12:i:-	RDNC	AMP,STR,SMX,TET
395/14	Meat product	Derby 4,12:f,g:-	ND	STR,SMX,TET
396/14	Meat product	Derby 4,12:f,g:-	ND	STR,SMX,TET
399/14	Meat product	Typhimurium 4,12:i:1,2	PT195	AMP,NAL,TET
400/14	Meat product	Rissen 6,7:fg:-	ND	AMP,CHL,SMX,TET,TMP,AZM
401/14	Meat product	Rissen 6,7:fg:-	ND	AMP,CHL,NAL,SMX,TET,TMP,AZM
402/14	Meat product	Rissen 6,7:fg:-	ND	AMP,CHL,GEN,SMX,TET,TMP,AZM
403/14	Meat product	Rissen 6,7:fg:-	ND	AMP,CHL,NAL,SMX,TET,TMP,AZM

404/14	Meat product	Rissen 6,7:fg:-	ND	AMP,CHL,NAL,SMX,TET,TMP,AZM
405/14	Meat product	Typhimurium 4,5,12:i:-	U311	TET
406/14	Raw marinated meat	Typhimurium 4,5,12:i:-	UT	TET
407/14	Raw marinated meat	Typhimurium 4,5,12:i:-	U311	TET
408/14	Raw marinated meat	Typhimurium 4,5,12:i:-	U311	TET
419/14	Raw marinated meat	Typhimurium 4,5,12:i:-	U311	SMX,TET
449/14	Raw marinated meat	Typhimurium 4,5,12:i:1,2	RDNC	AMP
456/14	Shellfish	Typhimurium 4,5,12:i:1,2	RDNC	TET
457/14	Shellfish	Rissen 6,7:fg:-	ND	NAL,TET,TMP
498/14	Meat product	Typhimurium 4,12:i:-	PT138	AMP,STR,SMX,TET
29/15	Porcine carcass surface	London 3,10:I,v:1,6	ND	AMP,CHL,SMX,TET,TMP
30/15	Porcine carcass surface	London 3,10:I,v:1,6	ND	AMP,CHL,SMX,TET,TMP
31/15	Porcine carcass surface	Derby 4,12:f:g:-	ND	STR
32/15	Porcine carcass surface	Derby 4,12:f:g:-	ND	STR
33/15	Porcine carcass surface	Derby 4,12:f:g:-	ND	S
34/15	Porcine carcass surface	London 3,10:I,v:1,6	ND	AMP,CHL,SMX,TET,TMP
35/15	Porcine carcass surface	Typhimurium 4,12:i:1,2	U302	AMP,CHL,CIP, NAL, SMX,TET
36/15	Porcine carcass surface	Typhimurium 4,12:i:-	PT195	AMP, TET
37/15	Porcine carcass surface	Typhimurium 4,12:i:-	PT195	AMP, TET
38/15	Porcine carcass surface	Typhimurium 4,12:i:-	PT195	AMP,TET
39/15	Poutry product	Typhimurium 4,12:i:1,2	U302	AMP,CHL, SMX,TET
40/15	Porcine carcass surface	Typhimurium 4,12:i:1,2	U302	AMP,CHL,CIP,NAL, SMX,TET
55/15	Dry meat product	Typhimurium 4,12:i:-	PT195	AMP,TET
56/15	Dry meat product	Typhimurium 4,12:i:-	PT195	AMP,TET
57/15	Dry meat product	Typhimurium 4,12:i:-	PT195	AMP,TET
58/15	Red dry sausage	Enteritidis 9,12:g,m:-	21	NAL
59/15	Red dry sausage	Enteritidis 9,12:g,m:-	21	NAL
60/15	Red dry sausage	Enteritidis 9,12:g,m:-	21	NAL
61/15	Dry meat product	Typhimurium 4,5,12:i:-	RDNC	AMP,STR,SMX,TET
62/15	Dry meat product	Typhimurium 4,5,12:i:-	RDNC	AMP,STR,SMX,TET
63/15	Dry meat product	Typhimurium 4,5,12:i:-	RDNC	AMP,STR,SMX,TET

119/15	Porcine carcass surface	Derby 4,12:f:g:-	ND	STR,SMX,TET
120/15	Porcine carcass surface	Typhimurium 4,12:i:1,2	PT104	AMP,CHL,SMX,TET
121/15	Porcine carcass surface	Typhimurium 4,12:i:1,2	PT104A	AMP,CHL,SMX,TET
124/15	Porcine carcass surface	Rissen 6,7:f,g:-	ND	TET
125/15	Porcine carcass surface	Reading 4,5,12:e,h:1,5	ND	S
126/15	Bovine carcass surface	Typhimurium 4,12:i	RDNC	AMP,STR,SMX,TET
127/15	Bovine carcass surface	Typhimurium 4,12:i	RDNC	AMP,STR,SMX,TET
128/15	Bovine carcass surface	Typhimurium 4,5,12:i:1,2	RDNC	AMP,SMX,TET
129/15	Bovine carcass surface	Typhimurium 4,5,12:i:1,3	RDNC	AMP,SMX,TET
130/15	Porcine carcass surface	Rissen 6,7:f,g:-	ND	TET
131/15	Porcine carcass surface	Rissen 6,7:f,g:-	ND	TET
132/15	Porcine carcass surface	Rissen 6,7:f,g:-	ND	TET
133/15	Porcine carcass surface	Typhimurium 4,5,12:i:1,2	RDNC	AMP,CHL,SMX,TET
134/15	Porcine carcass surface	Typhimurium 4,12:i:1,2	PT120	AMP,CHL,SMX,TET
**136/15**	**Porcine carcass surface**	**Kedougou 13,23:i:l,w**	ND	**SMX,TET, COL**
137/15	Porcine carcass surface	Rissen 6,7:f,g:-	ND	TET
138/15	Porcine carcass surface	Rissen 6,7:f,g:-	ND	TET
151/15	Porcine carcass surface	Bovismorbificans 6,8:r:1,5	ND	S
153/15	Porcine carcass surface	Typhimurium 4,12:i:-	PT138	AMP,STR,SMX,TET
154/15	Porcine carcass surface	Rissen 6,7:f,g:-	ND	TET
183/15	Fresh eggs	Typhimurium 4,12:i:-	RDNC	AMP,STR,SMX,TET
232/15	Minced meat	Typhimurium 4,12:i:-	PT195	AMP,STR,SMX,TET
233/15	Minced meat	Typhimurium 4,12:i:-	PT195	AMP,STR,SMX,TET
234/15	Minced meat	Typhimurium 4,12:i:-	PT195	AMP,STR,SMX,TET
235/15	Bovine carcass surface	Typhimurium 4,12:i:-	ND	AMP,STR,TET
236/15	Bovine carcass surface	Typhimurium 4,12:i:-	ND	AMP,STR,SMX,TET,TMP
**237/15**	**Pork and beef meat processed for raw consumption**	**Typhimurium 4,12:i:-**	PT104B	**AMP,STR,SMX,TET,COL**
238/15	Pork and beef meat processed for raw consumption	Typhimurium 4,12:i:-	PT104B	AMP,STR,SMX,TET
**295/15**	**Pork and beef minced meat**	**Typhimurium 4,5,12:i:-**	PT138	**AMP,STR,SMX,TET,COL**
296/15	Pork and beef minced meat	Typhimurium 4,5,12:i:-	PT138	AMP,STR,SMX,TET
297/15	Pork and beef minced meat	Typhimurium 4,5,12:i:-	PT138	AMP,STR,SMX,TET

**298/15**	**Pork and beef minced meat**	**Typhimurium 4,5,12:i:-**	RDNC	**AMP,STR,SMX,TET,COL**
299/15	Pork and beef minced meat	Typhimurium 4,5,12:i:-	PT138	AMP,STR,SMX,TET
313/15	Pork and beef minced meat	Typhimurium 4,5,12:i:-	PT138	AMP,STR,SMX,TET
314/15	Pork and beef minced meat	Typhimurium 4,5,12:i:-	PT138	AMP, STR,SMX,TET
315/15	Pork and beef minced meat	Typhimurium 4,5,12:i:-	PT138	AMP,STR,SMX,TET
316/15	Pork and beef minced meat	Typhimurium 4,5,12:i:-	PT138	AMP,STR,SMX,TET
317/15	Pork and beef minced meat	Typhimurium 4,5,12:i:-	PT138	AMP,STR,SMX,TET
318/15	Fresh meat product	Derby 4,12:f,g:-	ND	NAL,STR,SUL,TET
319/15	Minced meat	Typhimurium 4,5,12:i:-	U302	AMP,AMX,CHL,GEN,STR,TET
320/15	Minced meat	Typhimurium 4,5,12:i:-	PT22	AMP,AMX,CHL,GEN,STR,TET
321/15	Minced meat	Typhimurium 4,5,12:i:-	PT22	AMP,AMX,CHL,GEN,STR,TET
322/15	Minced meat	Typhimurium 4,5,12:i:-	PT22	AMP,AMX,CHL,GEN,STR,TET
323/15	Minced meat	Typhimurium 4,5,12:i:-	PT22	AMP,AMX,CHL,GEN,STR,TET
348/15	Wild boar minced meat	Typhimurium 4,5,12:i:-	RDNC	AMP,STR,SMX,TET
349/15	Wild boar minced meat	Typhimurium 4,5,12:i:-	RDNC	AMP,STR,SMX,TET
350/15	Wild boar minced meat	Typhimurium 4,5,12:i:-	PT138	AMP,STR,SMX,TET
351/15	Wild boar minced meat	Typhimurium 4,5,12:i:-	PT138	AMP,CIP(I)-STR,SMX,TET
352/15	Wild boar minced meat	Typhimurium 4,5,12:i:-	RDNC	AMP,STR,SMX,TET
353/15	Wild boar minced meat	Typhimurium 4,5,12:i:-	RDNC	AMP,STR,SMX,TET
354/15	Wild boar minced meat	Typhimurium 4,5,12:i:-	RDNC	AMP,STR,SMX,TET
355/15	Wild boar minced meat	Typhimurium 4,5,12:i:-	RDNC	AMP,CHL,STR,SMX,TET
356/15	Wild boar minced meat	Typhimurium 4,5,12:i:-	RDNC	AMP,GEN,STR,SMX,TET
357/15	Wild boar minced meat	Typhimurium 4,5,12:i:-	PT138	AMP,CIP(I),STR,SMX,TET
358/15	Wild boar minced meat	Typhimurium 4,5,12:i:-	PT138	AMP,CIP(I),STR,SMX,TET
359/15	Wild boar minced meat	Typhimurium 4,12:i:-	PT138	AMP,CIP(I),STR,SMX,TET
360/15	Wild boar minced meat	Typhimurium 4,12:i:-	PT138	AMP,STR,SMX,TET
361/15	Wild boar minced meat	Typhimurium 4,5,12:i:-	RDNC	AMP,STR,SMX,TET
362/15	Wild boar minced meat	Typhimurium 4,5,12:i:-	RDNC	AMP,STR,SMX,TET
9/16	Red dry sausage	Rissen 6,7:fg:-	ND	AMP,GEN,TET
17/16	Porcine carcass surface	Typhimurium 4,12:i:1,2	RDNC	S
81/16	Bovine carcass surface	Derby 4,12:f,g:-	ND	S

82/16	Minced meat	Derby 4,12:f,g:-	ND	STR
83/16	Minced meat	Derby 4,12:f,g:-	ND	S
84/16	Fresh sausage	Typhimurium 4,12:i:-	NT	AMP
85/16	Fresh sausage	Typhimurium 4,12:i:-	ND	AMP
86/16	Fresh sausage	Typhimurium 4,12:i:-	PT138	AMP
87/16	Fresh sausage	Typhimurium 4,12:i:-	PT104b	AMP
88/16	Minced meat	Typhimurium 4,12:i:-	ND	AMP,STR,SMX
89/16	Minced meat	Goldcoast 6,8:r.l,w	RDNC	AMP,SMX,TET,TMP
90/16	Chorizo hash	Typhimurium 4,5,12:i:-	NT	AMP,STR,SMX,TET
91/16	Minced meat	Derby 4,12:f,g:-	ND	S
92/16	Porcine carcass surface	Typhimurium 4,12:i:-	195	STR
93/16	Porcine carcass surface	Typhimurium 4,5,12:i:-	NT	AMP,GEN,STR,SMX,TET,TOB
94/16	Porcine carcass surface	Rissen 6,7:fg:-	NT	CHL
101/16	Minced meat	Derby 4,12:f,g:-	ND	NAL
102/16	Minced meat	Derby 4,12:f,g:-	ND	S
103/16	Minced meat	Derby 4,12:f,g:-	ND	S
197/16	Bovine carcass surface	Typhimurium 4,12:i:-	PT195	AMP,STR,SMX,TET
267/16	Fresh egg	Enteritidis 9,12:g,m:-	NT	S
268/16	Fresh egg	Enteritidis 9,12:g,m:-	NT	S
269/16	Fresh egg	Enteritidis 9,12:g,m:-	PT34	S
270/16	Fresh egg	Enteritidis 9,12:g,m:-	NT	S
271/16	Eggshell	Enteritidis 9,12:g,m:-	NT	S
301/16	Eggshell	Enteritidis 9,12:g,m:-	PT59	S
320/16	Eggshell	Enteritidis 9,12:g,m:-	NT	S
321/16	Eggshell	Enteritidis 9,12:g,m:-	NT	S
323/16	Eggshell	Enteritidis 9,12:g,m:-	NT	S
324/16	Eggshell	Enteritidis 9,12:g,m:-	56	S
325/16	Eggshell	Enteritidis 9,12:g,m:-	NT	S
326/16	Fresh egg	Enteritidis 9,12:g,m:-	56	S
327/16	Fresh egg	Enteritidis 9,12:g,m:-	56	S
328/16	Spanish omelette	Enteritidis 9,12:g,m:-	56	S
66/17	Carcass surface	Enteritidis 9,12:g,m:-	56	ND

67/17	Marinated pork minced meat	Typhimurium 4,5,12:i:-	ND	AMP,STR,SMX,TET
68/17	Veal burger	Typhimurium 4,5,12:i:-	ND	AMP,STR,SMX,TET
69/17	Beef minced meat	Typhimurium 4,5,12:i:-	ND	AMP,STR,SMX,TET
124/17	Mollusk	Typhimurium 4,5,12:i:1,2	ND	ND
241/17	Spanish omelette	Enteritidis 9,12:g,m:-	ND	ND
242/17	Spanish omelette	Enteritidis 9,12:g,m:-	ND	ND
243/17	Spanish omelette	Enteritidis 9,12:g,m:-	ND	ND
244/17	Spanish omelette	Enteritidis 9,12:g,m:-	ND	ND
245/17	Spanish omelette	Enteritidis 9,12:g,m:-	ND	ND
1/18	UNK	Indiana 1,4,12:z:1,7	ND	ND
2/18	Carcass surface	Typhimurium 4,5,12:i:1,2	ND	AMP,CTX(I),KAN (I),STR,SMX,TET
3/18	Carcass surface	Typhimurium 4,5,12:i:1,2	ND	AMP,AMX(I),KAN(I),STR,SMX,TET,AZM
16/18	Minced meat	Typhimurium 4,12:i:1,2	ND	AMP,KAN,STR,SMX,TET
17/18	Minced meat	Typhimurium 4,5,12:i:1,2	ND	AMP,STR,SMX,TET,NIT(I)
42/18	Bovine carcass surface	Enteritidis 9,12:g,m:	ND	ND
**38/19**	**Fresh pork sausage**	**Typhimurium 4,5,12:i:-**	**ND**	**AMP,AMX,CTX,CHL,GEN,KAN,FOX,STR, SMX,TET,TMP,TOB, COL**
4/19	Porcine carcass surface	Derby 4,12:f,g:-	ND	S
5/19	Porcine carcass surface	Derby 4,12:f,g:-	ND	S
6/19	Chicken meat	Agona 4,12:f:,g,s:-	ND	S
217/19	Oysters	Enteritidis 9,12:g,m:-	ND	S

[a]Isolates are designated with the initials of the center which supplied them: "Laboratorio de Salud Pública" (LSP) of the Principality of Asturias, Spain, followed by a serial number/last two numbers of the year of recovery.

[b]Source relates to subsamples of analyzed samples. UNK, unknown source.

[c]PT, Phage Type (determined for isolates of serotypes Enteritidis, Typhimurium and the monophasic 4,[5],12:i:- variant until the middle of 2017; UT, UnTypeable; RDNC, Reacted but Did Not Conform.

[d] COL, colistin; AMP, ampicillin; AMX, amoxicillin- clavulanic acid, CTX, cefotaxime; FOX, cefoxitin; CHL, chloramphenicol; STR, streptomycin; GEN, gentamicin; TOB, tobramycin; KAN, kanamycin; SMX, sulfonamides; TET, tetracycline; TMP, trimethoprim; NIT, nitrofurantoin; AZM, azithromycin; NAL, nalidixic acid; CIP, ciprofloxacin; S, susceptible; I, intermediate.

Table S2. Accession numbers of the genomes of food-borne isolates of *Salmonella enterica* serovar 4,[5],12:i:- resistant to colistin, parameters related to the quality of the assemblies, as provided by VelvetOptimizer implemented in PLACNET (https://omictools.com/placnet-tool)

Isolate[a]	kmer	Contigs	N50	Longest contig (bp)	Total bp in contigs	Contigs > 1 kb	Library	Coverage	GenBank accession number
LSP 237/15	117	100	341,839	844,879	5,087,789	44	546 +/- 151	20x	JACXKV000000000
LSP 295/15	117	79	445,450	1,505,741	4,937,004	23	554 +/- 143	30x	JACXKU000000000
LSP 298/15	117	86	440,656	1,354,276	5,026,262	30	582 +/- 155	25x	JACXKT000000000
LSP 38/19	131	218	316,491	737,096	5,416,570	88	385 +/- 121	45x	JAGMWH000000000

[a], Isolates are designated with the initials of the center which supplied them: "Laboratorio de Salud Pública" (LSP) of the Principality of Asturias, Spain, followed by a serial number/last two numbers of the year of recovery.
